# Characterization of feeding damage by tea mosquito bug, *Helopeltis theivora* Waterhouse (Hemiptera: Miridae) on Hainan Dayezhong tea cultivar

**DOI:** 10.3389/fpls.2024.1529535

**Published:** 2025-01-07

**Authors:** Qi Yao, You-Ze Lin, Shuang Qin, Zhu-Feng Lin, Xun-Cong Ji

**Affiliations:** ^1^ Institute of Plant Protection, Hainan Academy of Agricultural Sciences, Haikou, China; ^2^ Research Center of Quality Safety and Standards for Agricultural Products of Hainan Academy of Agricultural Sciences, Haikou, China; ^3^ Key Laboratory of Plant Diseases and Pests of Hainan Province, Haikou, China

**Keywords:** tea mosquito bug, tea cultivar, Hainan Dayezhong tea, feeding behavior, feeding spot

## Abstract

The tea mosquito bug, *Helopeltis theivora* Waterhouse (Hemiptera: Miridae), is a devastating piercing-sucking pest in tropical tea plantations. The Hainan Dayezhong (HNDYZ) is a large-leaf tea cultivar widely cultivated around the Hainan tea region in South China. However, information regarding the feeding damage of *H. theivora* on the HNDYZ tea plant remains scarce. Here, we first describe the morphology of *H. theivora* in Hainan tea region. Subsequently, we investigate the feeding biology of *H. theivora* on HNDYZ tea shoots under laboratory conditions. Additionally, we survey the infestations of *H. theivora* in a small-leaf Jinxuan tea plantation and three large-leaf HNDYZ tea plantations under varying shaded conditions. The results indicated that the morphological features of eggs, nymphs, and adults of *H. theivora* in the Hainan tea region were similar to those of the same species reported in other tropical tea regions. Nymphs and adults of *H. theivora* primarily fed on tender leaves and produced a subcircular spot within 2 to 4 minutes. This feeding spot would gradually turn dark brown within 24 hours. Furthermore, the adjacent scattered spots would connect after 48 hours, resulting in a necrotic patch on the leaves by 72 hours. The peak feeding time for *H. theivora* occurred at night, specifically from 7:00 PM to 1:00 AM. The most preferred feeding site was at the second leaf position, accounting for 70.94 ± 3.68% of daily feeding spots. During the feeding peak, adults *H. theivora* produced more feeding spots than nymphs, with females and 5th-instar nymphs creating the largest feeding areas among all life stages. Field investigations showed that damage caused by *H. theivora* on the large-leaf HNDYZ tea cultivar was significantly greater than that on the small-leaf Jinxuan tea cultivar. More serious infestations of *H. theivora* were observed in the high-shade HNDYZ tea plantation compared to the medium-shade and no-shade HNDYZ tea plantations. This suggests that the different tea cultivars and shade conditions in tea plantations may influence the population of *H. theivora* in the field. These findings provide new insights for further research related to the feeding strategy of *H. theivora* on the HNDYZ tea cultivar.

## 1 Introduction

The tea mosquito bug, *Helopeltis theivora* Waterhouse 1886 (Hemiptera: Miridae) is a phytophagous pest that can damage a wide range of host plants, including crops, forestry trees, and several weeds that serve as alternate hosts in tropical countries (Roy et al., 2015; Saroj et al., 2016; Sankarganesh et al., 2020; Jakkoksung et al., 2023). This piercing-sucking insect pest typically feeds on the tender tissues of host plants. It produces several scattered brown spots quickly, resulting in quantitative and qualitative damage to crop production (Sankarganesh et al., 2020; Zhu, 2021). Tea plant (*Camellia sinensis*) is one of the most preferred hosts of *H. theivora*. Previous investigations indicated that *H. theivora* is widely distributed across the majority of tea plantations and has become a devastating pest for tea production in India, Bangladesh, Sri Lanka, Kenya, and several other tropical tea-producing countries in Asia and Africa (Hazarika et al., 2009; Ahmed and Mamun, 2014; Vishnupriya et al., 2021). Also, *H. theivora* is the primary and dominant pest throughout the tea regions in Hainan, the only tropicopolitan province of China (Meng et al., 2020; Zhou et al., 2023a).

The feeding damage caused by *H. theivora* on tea plants can result in a total loss of tea yield (Muraleedharan, 1992). When the adults and nymphs find suitable sites for feeding on tender tea shoots, they first insert their labial stylets into the epidermis of fresh buds, young stems, and leaves and then inject toxic saliva containing several digestive-related enzymes and finally suck the cell sap that has been digested (Roy et al., 2015; Sankarganesh et al., 2020). After infestation, a circular ring appeared around the feeding point on the leaf surface, while the area within the circle gradually discoloured to light brown within a few hours. Subsequently, the entire area turns dark brown and becomes an irregularly shaped spot that will become sunken and dry up within a few days (Vishnupriya et al., 2021; Radhakrishnan, 2022). These adjacent feeding spots will be elongated and connected to each other, resulting in the young leaves becoming deformed, curly, necrotic, and even defoliated (Sankarganesh et al., 2020).

Biological characteristics associated with the feeding behavior of *H. theivora* on tea plants have been extensively studied in laboratories and fields around tropical tea-producing countries (Roy et al., 2015). For instance, *H. theivora* preferred nocturnal feeding over diurnal feeding, with the number of feeding punctures during the night time being much greater than those during the daytime (Radhakrishnan, 2022). Moreover, the first and second leaves on the tea shoot were the most preferred positions for *H. theivora* feeding, with the percentage of spots accounting for more than 70% (Bhuyan and Bhattacharyya, 2006; Roy et al., 2009). In addition, the different instar nymphs and adults of *H. theivora* can create varying areas and quantities of feeding spots on tea leaves. The average diameters of these spots ranged from 0.29 mm to 2.57 mm (Bhuyan and Bhattacharyya, 2006), while the number of spots produced varied from 60.2 to 104.6 per individual per day (Roy et al., 2009). Generally, adults made more spots than nymphs.

The feeding behavior of tea mosquito bugs (*Helopeltis* spp.) on tea plants can be influenced by various abiotic and biotic factors. For instance, the tea cultivar is one of the most important factors, while the unique physical properties (e.g., color and tenderness) and chemical components (e.g., primary and secondary metabolites) of different tea shoots can mediate the probing attempts and feeding decisions of *H. theivora* (Roy et al., 2009; Chowdhury et al., 2016; Bordoloi et al., 2023; Borthakur and Bora, 2023; Samynathan et al., 2023). Simultaneously, the diverse micro-circumstances in tea plantations such as changes in temperature, rainfall intensity, photoperiod variation, and the use of intercropping and cover crops can affect the growth and development of tender shoots, which in turn influences the feeding preferences of *H. theivora* (Chakraborty and Chakraborty, 2005; Pakrashi and Sarma, 2014; Sarma et al., 2014; Babu et al., 2023). Additionally, the population of natural enemies in tea plantations, particularly some species in the two important predators, Reduviidae and Oxyopidae, for *H. theivora*, could also influence the extent of feeding damage caused by this pest (Basnet and Mukhopadhyay, 2014; Srikumar et al., 2017; Bharathi and Rabeesh, 2023).

Hainan Dayezhong (HNDYZ) is a large-leaf tea cultivar that has been identified as an independently originated cultivar, distinct from the Assam-type large-leaf tea in India and other large-leaf tea cultivars in mainland China (Guo et al., 2024). HNDYZ is currently the most widespread cultivar in the tea regions of Hainan province, China, with a total area of 1,325.2 hectares, accounting for 56.7% of all tea-producing areas in Hainan (Lu and Zhang, 2023). *H. theivora* is an important sucking pest on the HNDYZ tea plants, which occurs in the whole tea-growing seasons in Hainan (Meng et al., 2020). Feeding damage caused by *H. theivora* has led to significant losses in HNDYZ tea production in Hainan (Zhou et al., 2023a). Nevertheless, information on the feeding biology of *H. theivora* on the HNDYZ tea plant is still scarce. Meanwhile, there are three typical HNDYZ tea plantations in the Hainan tea region, categorized by the different shade conditions in the tea fields. The first type is the high-shade tea plantation, where HNDYZ tea plants are interplanted beneath tall, dense trees in the tropical rainforest (e.g. *Michelia balansae* and *Parakmeria lotungensis*), allowing only 20% of sunlight to reach the surface of the tea bushes. The second type is the medium-shade tea plantation, where tea plants are interplanted with betel trees (*Areca catechu*), allowing about half the sunlight to reach the tea canopy. The third type is the no-shade tea plantation, where tea plants are cultivated in bare lands without any intercropping (Wang, 1985; Guo et al., 2006; Peng et al., 2023). However, the difference in feeding damage caused by *H. theivora* in those three HNDYZ tea plantations remains unknown.

In this study, we addressed three pivotal questions to systematically demonstrate the feeding damage of *H. theivora* on the HNDYZ tea cultivar. (1) Are the morphological characteristics of *H. theivora* in the Hainan tea region similar to those reported in other tropical tea regions? (2) How are the performances associated with the feeding biology of *H. theivora* on HNDYZ tea plants? (3) What are the differences in feeding damage caused by *H. theivora* between large-leaf and small-leaf tea cultivars, as well as among the three HNDYZ tea plantations under different shade conditions? These attempts to emphasize the feeding activities of *H. theivora* on the large-leaf HNDYZ tea in the tropical region of South China will contribute to an in-depth study of the feeding strategies of tea mosquito bugs on HNDYZ tea plants.

## 2 Materials and methods

### 2.1 Insects and tea plants

Adults and Nymphs of *H. theivora* were captured in the HNDYZ tea plantations, which were located in the rural areas in Shuiman town, Wuzhishan city, Hainan province of China (18.89°N, 109.67°E). These *H. theivora* were subsequently reared in a woody insect-breeding cage (40 cm × 40 cm × 43 cm) in the laboratory with the hydroponic tea shoots of HNDYZ in the Institute of Plant Protection, Hainan Academy of Agricultural Sciences in Haikou, China. The temperature in the laboratory was maintained at 26 ± 2°C, while the relative humidity was kept at 70 ± 3% with the photoperiod at 12:12 hr (L:D). In addition, the tender shoots of tea plants used in this study were all clipped in the HNDYZ tea plantations in Shuiman, Wuzhishan, China.

### 2.2 Morphology of *H. theivora* in Hainan tea region

The morphological characteristics of *H. theivora* in HNDYZ tea plantation were detected using the camera (Canon 6d2 + EF 100mm f/2.8 IS USM, Japan) and the super depth of field 3D microscope (Keyence VHX-7000, Japan). The egg and the 1st-5th instar nymphs were investigated using the super depth microscope VHX-7000 at 150 times magnification. The adult male and female were directly recorded with the camera Canon 6d2. Moreover, the lengths of two unequal respiratory horns which were distributed on the egg operculum and the length of the egg without those two horns were measured with VHX-7000, respectively.

### 2.3 Variation tracking for feeding spots of *H. theivora* on HNDYZ tea leaves

The super depth microscope VHX-7000 was used to monitor the dynamic changes of feeding spots made by *H. theivora* on tea leaves. A 3rd-instar nymph of *H. theivora* was selected for feeding on the 2nd-leaf position of HNDYZ tea shoot. When the nymph finished producing four adjacent spots on the leaf, the infested leaf was immediately removed from the tea shoot and grown hydroponically above moistened floral foam under laboratory conditions (26 ± 2°C, 70 ± 3% RH, and 12L:12D). Thereafter, the transformation of those feeding spots were recorded by using the VHX-7000 at 50 times magnification at 1 hr, 2 hr, 3 hr, 5 hr, 7 hr, 14 hr, 24 hr, 48 hr, 72 hr, and 96 hr, respectively.

### 2.4 Detection of each feeding process for *H. theivora* on HNDYZ tea leaves

This experiment was designed to depict the feeding process for *H. theivora* on the tender leaves of HNDYZ tea and test whether there was a significant difference in the duration of each feeding spot between the nymphs and adults. The camera Canon 6d2 was used to record the whole feeding process for each *H. theivora*. The duration of each feeding spot was defined as beginning when the labial stylets punctured the leaf epidermis and ending when the labial stylets were withdrawn from the leaf surface. Ten adults and ten 3rd-instar nymphs of *H. theivora* were investigated, respectively. Four durations were recorded and averaged for each individual, and the mean values were used for the significance comparison between nymphs and adults.

### 2.5 Determinations of feeding time and place of *H. theivora* on HNDYZ tea plants

The 3rd-instar nymph was used to evaluate the daily feeding peak and the most preferred feeding sites for *H. theivora* on the HNDYZ tea shoots. Twelve time-regimes were designed within a whole day at 1:00 ~ 3:00, 3:00 ~ 5:00, 5:00 ~ 7:00, 7:00 ~ 9:00, 9:00 ~ 11:00, 11:00 ~ 13:00, 13:00 ~ 15:00, 15:00 ~ 17:00, 17:00 ~ 19:00, 19:00 ~ 21:00, 21:00 ~ 23:00, and 23:00 ~ 1:00, respectively. Four 3rd-instar nymphs were introduced for feeding on 3 tender shoots of HNDYZ for 2 hours of each time interval. The infested shoots were fetched out, and another 3 fresh tender shoots of HNDYZ were replaced. The number of feeding spots was counted consecutively every 2 hours a day. Simultaneously, the feeding spots on the buds, the 1st leaf, the 2nd leaf, and the 3rd leaf were also calculated, respectively. Three biological replications were implemented in this experiment.

### 2.6 Determinations of amount and area of feeding spots by *H. theivora* on HNDYZ tea plants

This experiment was conducted to distinguish the quantity and the area of feeding spots produced by *H. theivora* at different life history stages. The 1st-instar nymph, 2nd-instar nymph, 3rd-instar nymph, 4th-instar nymph, 5th-instar nymph, and adults male and female were tested, respectively. Ten *H. theivora* were introduced into a cloth insect cage (30 cm × 30 cm × 30 cm) for feeding on 10 tender shoots of HNDYZ tea cultivar for 6 hours during the feeding peak at night time (i.e. 7:00 PM to 1:00 AM). Thereafter, the infested shoots were taken out, and the number of feeding spots produced by ten *H. theivora* was counted for the significance test. Four biological replications were conducted, and significance tests were performed for *H. theivora* among different instar nymphs, as well as adult males and females, respectively. In addition, the areas of each feeding spot produced by the 1st-instar nymph, 2nd-instar nymph, 3rd-instar nymph, 4th-instar nymph, 5th-instar nymph, and adults male and female were measured, respectively, by using the VHX7000 for irregular images at 20 times magnification. Ten feeding spots were identified for *H. theivora* at each life stage for the significance comparison.

### 2.7 Investigations of feeding damage of *H. theivora* in different tea plantations

**Figure 1:**
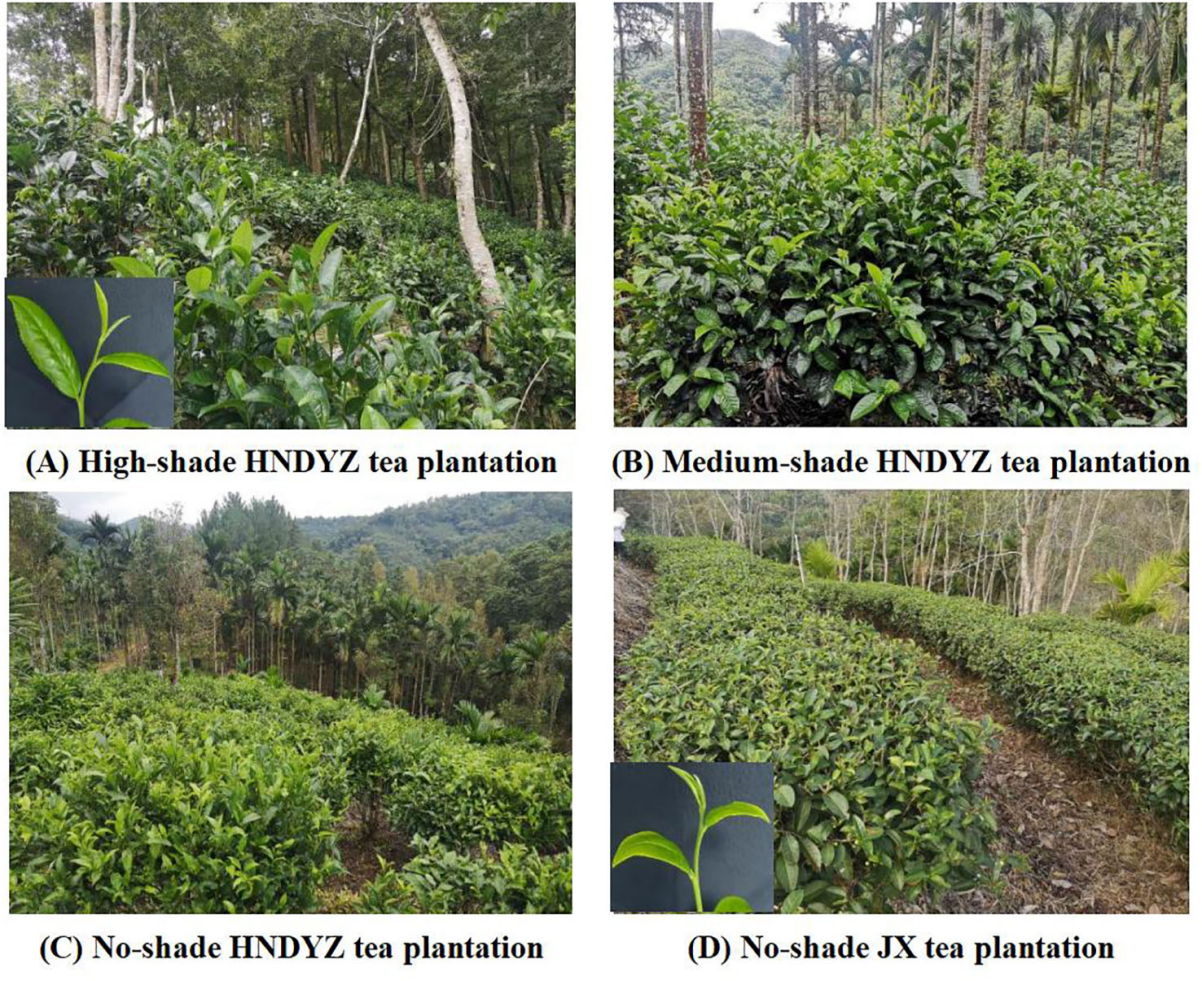
Four types of tea plantation in Hainan, China. There are three typical large-leaf Hainan Dayezhong (HNDYZ) tea plantations and one small-leaf Jinxuan (JX) tea plantation. **(A)**: The first type is the high- shade tea plantation that HNDYZ tea plants are cultivated beneath the tall and dense trees (e.g. tropical rainforest), which allows about 20% sunlight to reach the tea bushes. **(B)**: The second type is the medium-shade tea plantation that HNDYZ tea plants are interplanted with the rubber or betel trees, which allows about half sunlight to reach the tea canopy. **(C)**; The third type is the no-shade tea plantation that HNDYZ tea plants are cultivated at the bare land without any intercropping. **(D)**: The no-shade JX tea plantation that JX tea plants are cultivated at the bare land without any intercropping. The pictures of tender shoots (one bud and two leaves) of HNDYZ and JX were inset in the picture **(A, D)**, respectively.

A field investigation was conducted to determine whether different tea cultivars and shade conditions in tropical tea plantations affect the damage caused by *H. theivora* in Hainan, China. We surveyed the feeding damage caused by *H. theivora* in three large-leaf HNDYZ tea plantations and one small-leaf Jinxuan (JX) tea plantation during the peak occurrence in August 2023. The three HNDYZ tea plantations included high-shade, medium-shade, and no-shade tea plantations, respectively (**Figure 1**). The five-point sampling method was used for field surveys, with survey points established in different directions: one at the center of the tea plantation and others to the east, west, south, and north. One hundred harvestable tender shoots were examined, and the number of shoots with one bud and two leaves affected by feeding damage from *H. theivora* was recorded. Five biological replications were implemented for each survey point. The significance tests were conducted between the HNDYZ and JX tea plantations, as well as among the high-shade, medium-shade, and no-shade HNDYZ tea plantations.

### 2.8 Data analysis

Data were processed and analyzed using the statistical software SPSS (Version 25.0). The data normality was first tested by using the Shapiro-Wilk method. The variance homogeneity for all the data was estimated according to the Levene test. The normally distributed data were subjected to ANOVA analysis, followed by the Tukey HSD *post hoc* test, while the non-normally distributed data were addressed by the Kruskal-Wallis test. Accordingly, the daily feeding rhythm, the preferred feeding sites, the number of feeding spots at different life stages, and the feeding damage in different tea plantations were analyzed using the one-way ANOVA with Tukey HSD *post hoc* test. The acreage of each feeding spot produced by *H. theivora* at different life stages was analyzed with the Kruskal-Wallis test. Additionally, the duration of each feeding spot produced between nymph and adult were subjected to the independent sample *t-test*. All the statistical figures were plotted using GraphPad Prism (Version 8.0.1).

## 3 Results

### 3.1 Morphological characteristics of *H. theivora* in Hainan tea region

**Figure 2:**
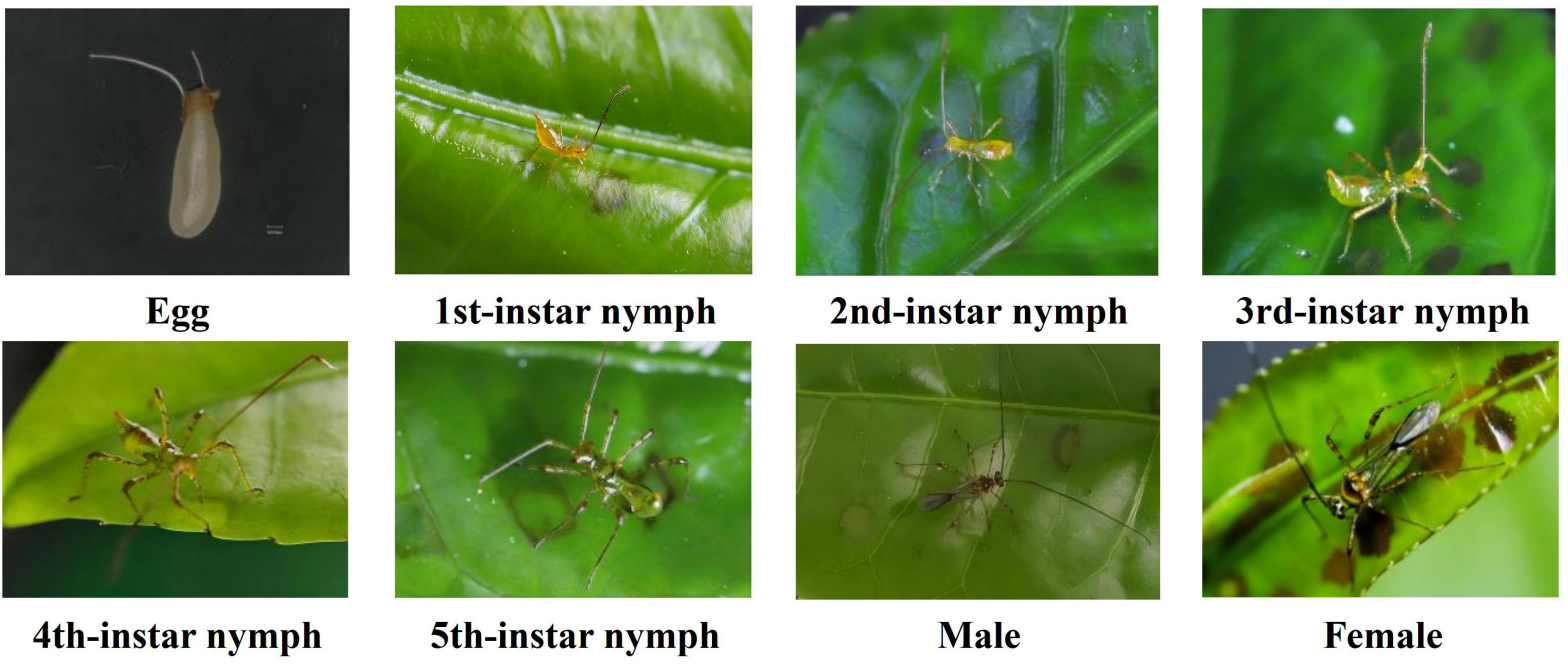
Morphological characteristics of *Helopeltis theivora* in Hainan tea region. The nymphs and adults of *H. theiora* were all captured in the HNDYZ tea plantations, which were located in Shuiman town, Wuzhishan city, Hainan province of China (18.89°N, 109.67°E). Particularly, the egg was detached from tender stem and was investigated in laboratory by using the super depth microscope VHX-7000 (Keyence, Japan) with 150 times magnification.

There were three stages in the life history of *H. theivora* in HNDYZ tea plantations: the egg, nymphal, and adult (**Figure 2**). The egg of *H. theivora* initially emerged in a sausage shape and was a milky white color at the time of oviposition. It then gradually transformed to a light yellow and ultimately exhibited a nacarat color as it approached incubation. *H. theivora* eggs were mainly deposited below the epidermic of tender stem with a length of 1049.70 ± 36.32(SE) μm (n=6). There were two unequal chorionic processes on the egg operculum vertically elongated outside the plant surface, with the longer process above the shorter one (Personal observation). The length of the longer process (651.00 ± 21.74 μm, n=6) was more than twice as the shorter one (311.90 ± 21.01 μm, n=6). There were five instars during the nymphal stage of *H. theivora*. The body color of the freshly hatched 1st-instar nymph is nacarat, followed by yellow-green for the 2nd and 3rd-instar nymphs, green for the 4th-instar nymph, and dark green for the 5th-instar nymph, respectively (**Figure 2**). The body size and color of the pronotum differed significantly between adult male and female *H. theivora*. The female was much larger than the male. Interestingly, the pronotum of the female exhibited a yellowish-brown color, while that of the male was usually dark brown (**Figure 2**).

### 3.2 Variation of feeding spots of *H. theivora* on HNDYZ tea leaves

**Figure 3:**
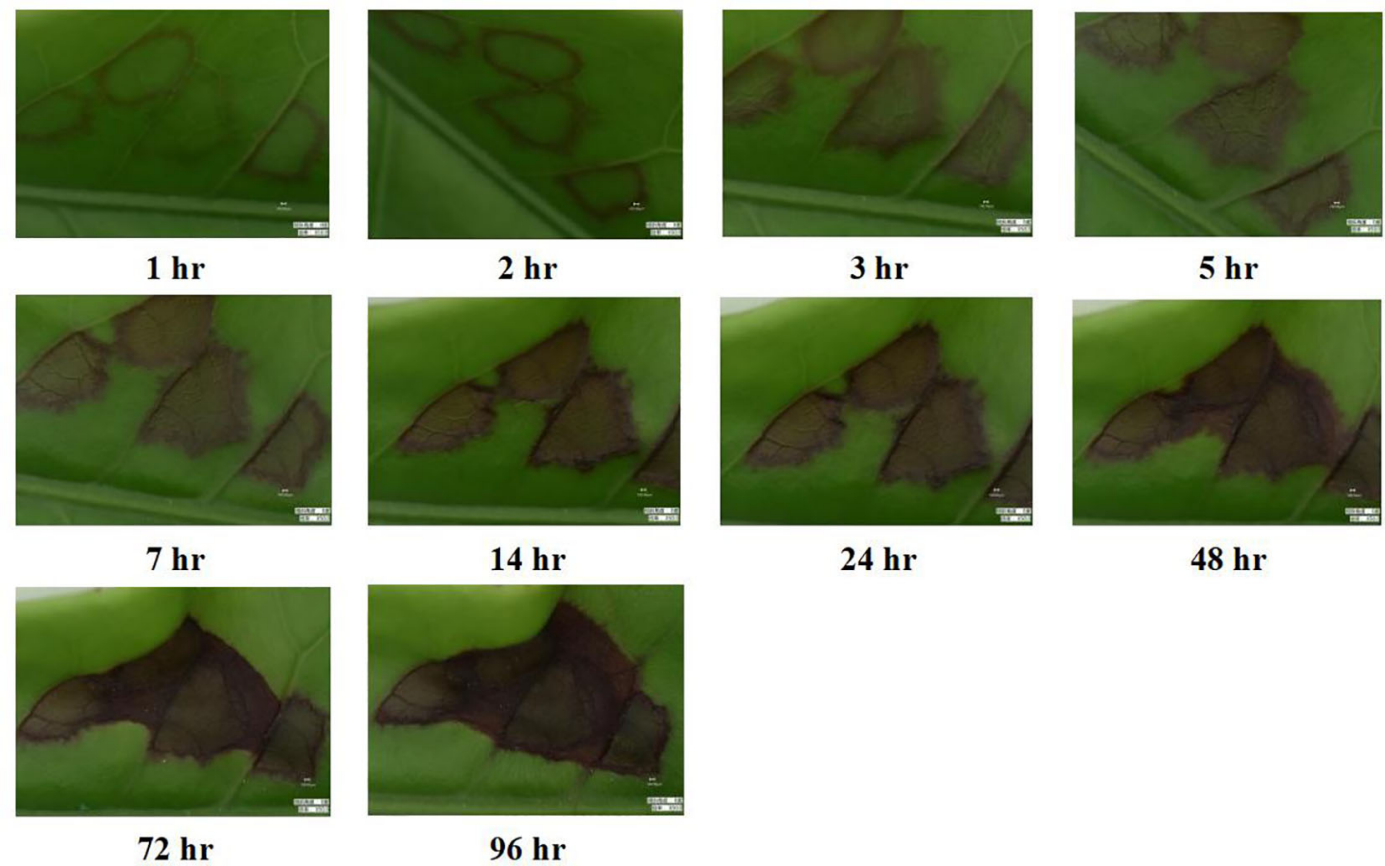
Variation of feeding spots produced by *Helopeltis theivora* on tender leaf of HNDYZ tea cultivar. The 2nd-leaf of HNDYZ tea shoot was fed by a 3rd-instar nymph of *H. theivora* and the feeding spots were investigated after being infested in 0.5 hr, 1 hr, 3 hr, 5 hr, 7 hr, 14 hr, 24 hr, 48 hr, 72 hr, and 96 hr, respectively, by using the super depth microscope VHX-7000 (Keyence, Japan) with 50 times magnification.

**Figure 4:**
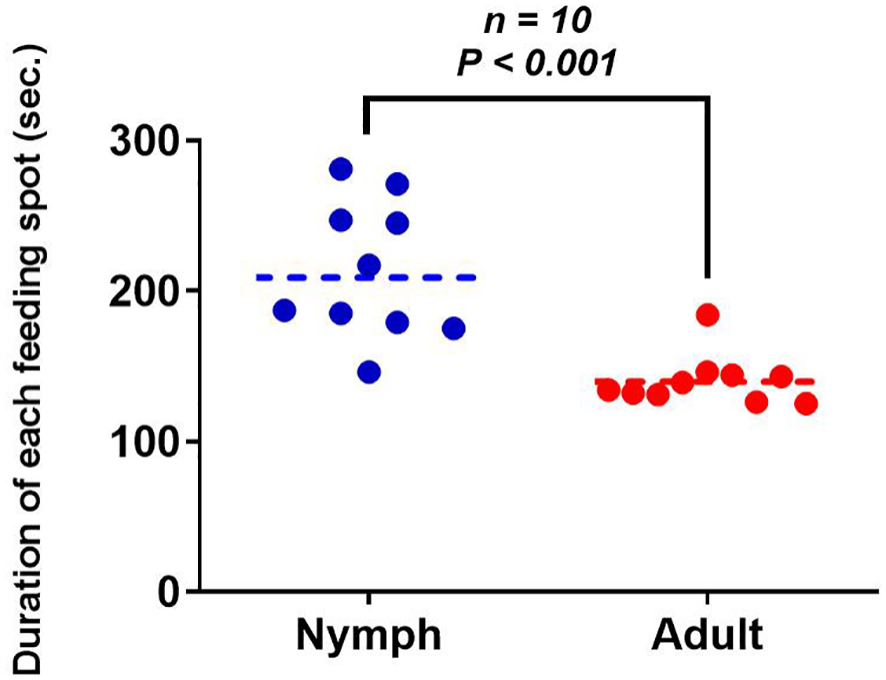
Duration of each spot produced by nymphs and adults of *Helopeltis theivora* on HNDYZ tea shoots. Data are the Means ± SE. The ten 3rd-instar nymphs and ten adults of *H. theivora* were tested, respectively. The duration of each feeding spot was recorded for each nymph and adult, and four biological replications were implemented per individual. The significance test was performed by using the Independent sample t-test at P<0.05.

After the 3rd-instar nymph of *H. theivora* penetrated the leaf surface and began to suck the sap, a slight circular spot appeared around the puncture point on the leaf within 1 hour (**Figure 3**). Thereafter, the circle quickly turned dark, and the inner area gradually developed a light brown color within 2 to 3 hours, starting from the edge and moving toward the center due to the host plant’s hypersensitive response (**Figure 3**). The entire spot on the leaf became dark brown within 24 hours after being infested by *H. theivora*. This was followed by merging several adjacent spots within 48 hours, ultimately leading to leaf drying and necrosis within 96 hours (**Figure 3**). Interestingly, there was a significant difference in the duration of each feeding spot produced by the nymphs and adults of *H. theivora* (*t* = 4.735, *df* = 18, *P* < 0.001). The feeding duration for each spot by adults was 140.40 ± 5.37 sec., which was noticeably faster than that by the nymphs with 213.30 ± 14.43 sec. (**Figure 4**).

### 3.3 Daily feeding rhythm of *H. theivora* on HNDYZ tea shoots

**Figure 5:**
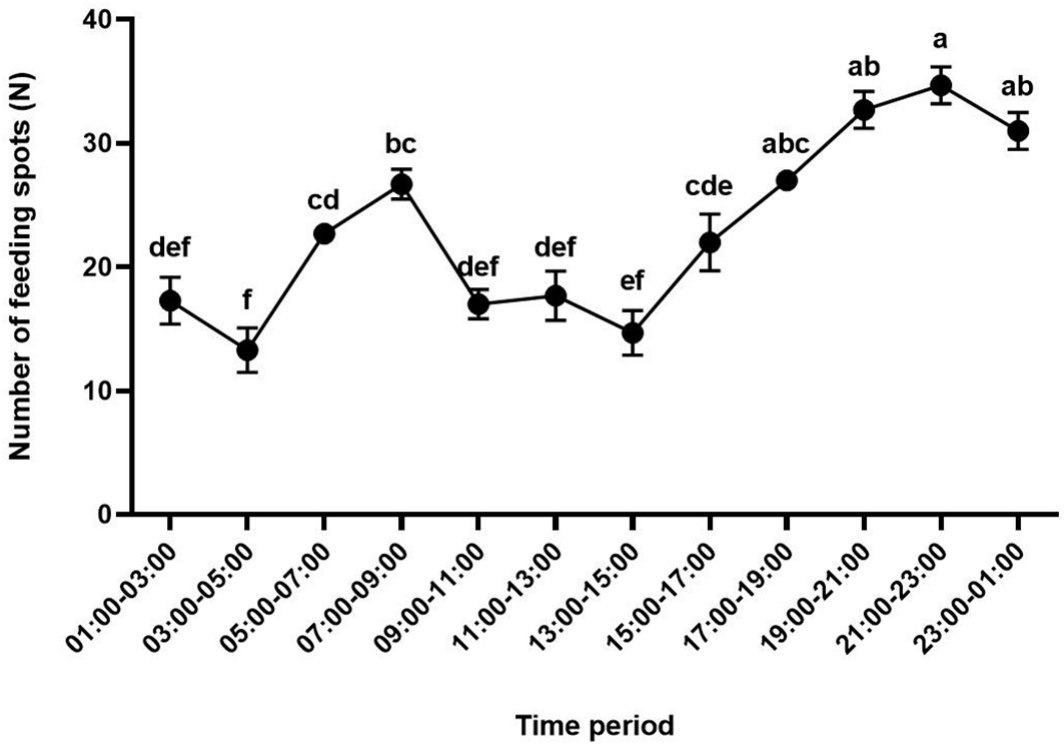
Daily feeding rhythm of *Helopeltis theivora* on HNDYZ tea plants. Data are the Means ± SE. The number of feeding spots produced by four 3rd-instar nymphs of *H. theivora* was investigated in every two hours in an consecutive 24 hr in laboratory, and three biological replications were implemented. Different letters above the number of feeding spots indicate a significant difference between two different time intervals in a day at *P*<0.05 (ANOVA, Tukey).

The feeding spots on the tea leaves appeared at each time interval, indicating that the feeding behavior of *H. theivora* on the HNDYZ tea cultivar occurred throughout the day (**Figure 5**). However, there was a distinct rhythm for *H. theivora* feeding daily on tea leaves (*F* = 22.035, *df_1_* = 11, *df_2_* = 24, *P* < 0.001) with two feeding peaks exhibiting at the periods from 5:00 PM to 1:00 AM and from 5:00 AM to 9:00 AM, respectively (**Figure 5**). Therein, four 3rd-instar nymph of *H. theivora* produced the maximum feeding amount with the 34.7 ± 1.5 spots between 9:00 PM and 11:00 PM, which showed no significant difference to the 32.7 ± 1.5 spots produced between 7:00 PM and 9:00 PM, the 31.0 ± 1.5 spots produced between 11:00 PM and 1:00 AM, and the 27.0 ± 0.6 spots produced between 5:00 PM and 7:00 PM, but was significantly higher than those produced at the other periods in a whole day, suggesting a primary feeding peak from 5:00 PM to 1:00 AM for *H. theivora* on HNDYZ tea shoots (**Figure 5**). The following feeding amount resulted in 26.7 ± 1.2 spots produced between 7:00 AM and 9:00 AM, which showed no significant difference compared to the 22.7 ± 0.3 spots produced between 5:00 AM and 7:00 AM. However, both amounts were significantly higher than those recorded during the remaining periods. This suggests a second feeding peak for *H. theivora* on HNDYZ tea shoots from 5:00 AM to 9:00 AM (**Figure 5**). Conversely, the minimum feeding amount was 13.3 ± 1.8 spots produced between 3:00 AM and 5:00 AM, which showed no significant differences compared to the spots produced between 1:00 AM and 3:00 AM, between 9:00 AM and 11:00 AM, between 11:00 AM and 1:00 PM, and between 1:00 PM and 3:00 PM, respectively. This indicates that both the mid-day period from 9:00 AM to 3:00 PM and the late-night period from 1:00 AM to 5:00 AM were not the habitual feeding times for *H. theivora* on the HNDYZ tea plants (**Figure 5**).

### 3.4 Preferred feeding sites of *H. theivora* on HNDYZ tea shoots

**Figure 6:**
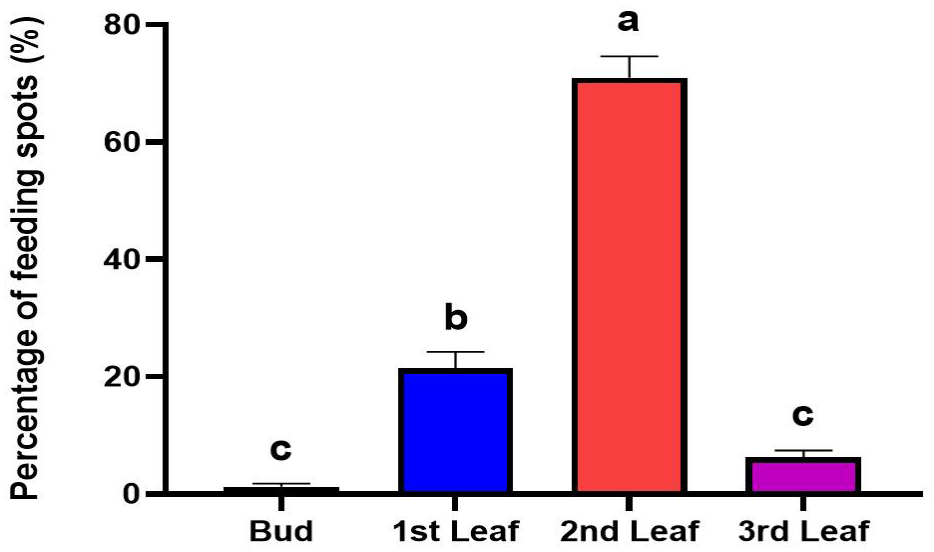
Feeding sites of *Helopeltis theivora* on HNDYZ tea plants. Data are the Means ± SE. The percentage of feeding spots produced by four 3rd-instar nymphs of *H. theivora* at four sites of HNDYZ tea shoots was calculated for 24 hr in laboratory, and three biological replications were implemented. Different letters above the percentage of feeding spots indicate a significant difference between two different leaf positions on a tea shoot at *P*<0.05 (ANOVA, Tukey).

The feeding positions of *H. theivora* on HNDYZ tea shoots included the bud, the 1st-leaf, the 2nd-leaf, and the 3rd-leaf, suggesting a feeding preference for tenderness (**Figure 6**). The maximum feeding amount was observed at the 2nd leaf, with a percentage of 70.94 ± 3.68%. This was significantly higher than the amounts recorded at other locations, indicating that the 2nd leaf was the most preferred site for feeding by *H. theivora* on the HNDYZ tea shoot (**Figure 6**). The feeding amount observed at the 1st leaf was 21.51 ± 2.70%, significantly higher than those at the 3rd leaf and the bud, which were 6.30 ± 1.12% and 1.25 ± 0.50%, respectively. The minimum feeding amounts were recorded at the 3rd leaf and the bud, with no significant difference (**Figure 6**).

### 3.5 Feeding amount of nymphs and adults of *H. theivora* on HNDYZ tea shoots

**Figure 7:**
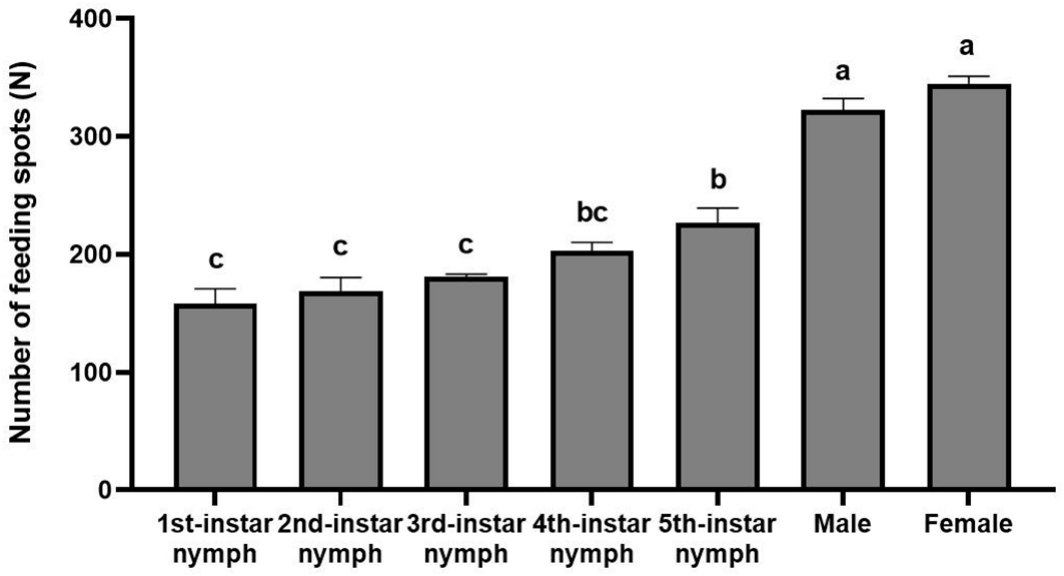
Feeding amount of nymphs and adults of *Helopeltis theivora* on HNDYZ tea shoots. Data are the Means ± SE. The number of feeding spots produced by ten different instar nymphs and adults male and female of *H. theivora* was recorded for 6 hr during the feeding peak (7:00 PM to 01:00 AM) in laboratory, and four biological replications were implemented. The significance test was performed with ANOVA, followed by the Tukey HSD post hoc test. Different letters above the number of feeding spots indicate a significant difference between two treatments at *P*<0.05.

There was a significant difference in the feeding amounts among the various instar nymphs and adult males and females of *H. theivora* on the HNDYZ tea leaves (*F* = 57.565, *df_1_* = 6, *df_2_* = 21, *P* < 0.001). During the feeding peak from 7:00 PM to 1:00 AM, the ten females produced 344.3 ± 6.8 spots on tea leaves, which was not significantly different from the 322.3 ± 9.9 spots produced by the males. However, this was significantly higher than the number of spots produced by nymphs from the 1st instar to the 5th instar, suggesting that adults *H. theivora* fed more than the nymphs (**Figure 7**). The ten 5th-instar nymphs of *H. theivora* could produce 226.8 ± 12.5 spots over 6 hours, from 7:00 PM to 1:00 AM. This number showed no significant difference compared to the 202.5 ± 7.7 spots produced by the 4th-instar nymphs, but was significantly higher than the 180.8 ± 2.4 spots produced by the 3rd-instar nymphs, the 168.3 ± 12.1 spots produced by the 2nd-instar nymphs, and the 157.8 ± 13.1 spots produced by the 1st-instar nymphs, respectively (**Figure 7**). The results indicated that the feeding amount of *H. theivora* gradually increased with body growth and development.

### 3.6 Feeding area of nymphs and adults of *H. theivora* on HNDYZ tea shoots

**Figure 8:**
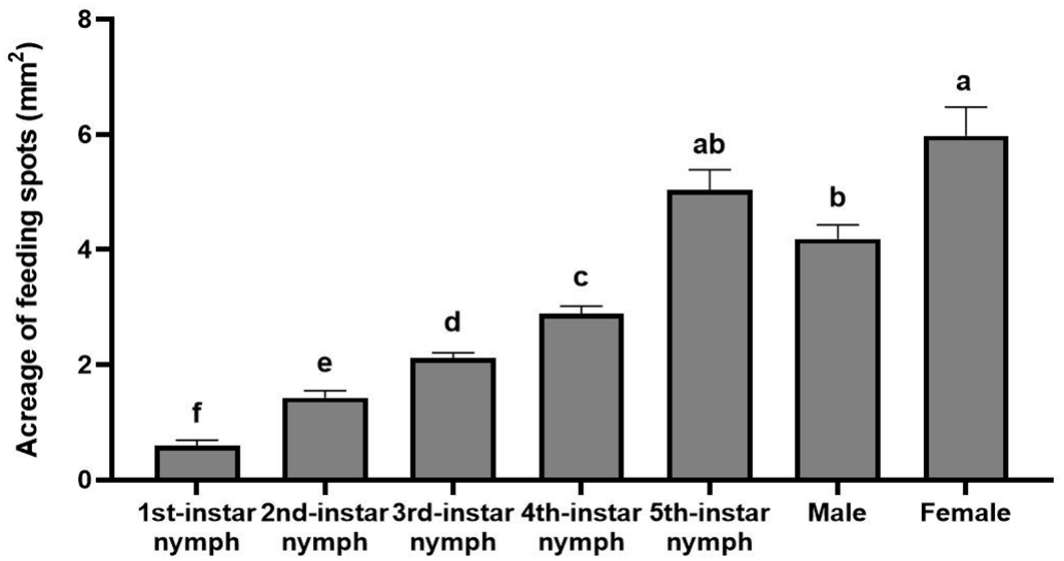
Feeding area of nymphs and adults of *Helopeltis theivora* on HNDYZ tea shoots. Data are the Means ± SE. The acreage of each feeding spot produced by different instar nymphs and adults male and female of *H. theivora* was measured, respectively, by using the super depth microscope VHX-7000 (Keyence, Japan) with irregular images at 20 times magnification: Ten spots were measured per individual and the mean value was calculated. The significance test was performed by using the Kruskal-Wallis test, which was followed by the Multiple comparisons with the Stepwise step-down. Different letters above the acreage of feeding spots indicate a significant difference between two treatments at *P*<0.05.

The feeding spots produced by the different instar nymphs and the adult male and female of *H. theivora* also showed a significant difference (Kruskal-Wallis test: *χ^2^* = 63.126, *df* = 6, *P* < 0.001). Therein, the largest quantitative spot was produced by the adult female with an area of 5.98 ± 0.50 mm^2^, which exhibited no significant difference to the spot produced by the 5th-instar nymph with an area of 5.04 ± 0.35 mm^2^ but was significantly larger than that produced by the males and the other instar nymphs, suggesting that the female and 5th-instar nymph were the most destructive stages during the life history (**Figure 8**). The following areas of feeding spots were produced by the adult male with 4.19 ± 0.24 mm^2^, the 4th-instar nymph with 2.84 ± 0.13 mm^2^, the 3rd-instar nymph with 2.12 ± 0.09 mm^2^, the 2nd-instar nymph with 1.43 ± 0.12 mm^2^, and the 1st-instar nymph with 0.59 ± 0.10 mm^2^, respectively, which all presented the significant differences between each other (**Figure 8**). Particularly, the significant differences observed between any two consecutive instar nymphs suggest a persistent increase in feeding damage to HNDYZ tea shoots, corresponding with the growth and development of *H. theivora* during the nymphal stage.

### 3.7 Feeding Damage of *H. theivora* in different plantations in Hainan tea region

**Figure 9:**
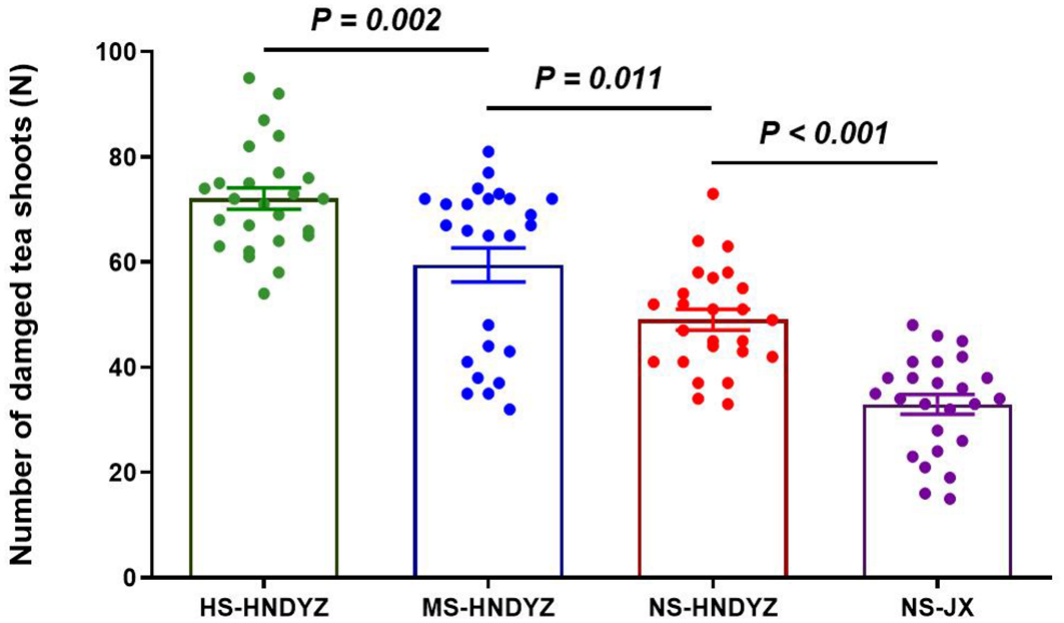
Feeding damage of *Helopeltis theivora* in different tea plantations in Hainan, China. Data are the Means ± SE. Field survey was conducted on August 29, 2023. The four tea plantations were all located in Shuiman, Wuzhishan, China (18.89°N, 109.67°E). Herein, there were three Hainan Dayezhong (HNDYZ) tea plantations including high-shade (HS-HNDYZ), medium-shade (MS-HNDYZ), and no-shade (NS-HNDYZ) tea plantations, and one no-shade Jinxuan (NS-JX) tea plantation. Five survey points were established in the different directions (i.e. centre, east, west, south, and north) inside each tea plantation. The number of damaged shoots of *H. theivora* per 100 shoots was investigated, respectively. Five biological replications were implemented for each survey point. Significance tests were conducted between the NS-HNDYZ and NS-JX tea plantations, as well as among the HS-HNDYZ, MS-HNDYZ and NS-HNDYZ tea plantations, with one-way ANOVA followed by the Turkey HSD post hoc test. The significant difference was identified between two treatments at *P*<0.05.

During the peak season in August 2023, there was a significant difference in the feeding damage caused by *H. theivora* between three large-leaf HNDYZ tea plantations and one small-leaf JX tea plantation (*F* = 50.086, *df_1_* = 3, *df_2_* = 96, *P* < 0.001). The number of damaged tea shoots by *H. theivora* in the no-shade HNDYZ tea plantation was 49.0 ± 2.0 per 100 shoots, which exhibited significantly higher than the 32.9 ± 1.9 damaged shoots in the no-shade JX tea plantation, suggesting that *H. theivora* preferred feeding on the large-leaf tea cultivar than on the small-leaf tea cultivar (**Figure 9**). Moreover, there was also a significant difference among three HNDYZ tea plantations under different shade conditions. Particularly, the high-shade HNDYZ tea plantation suffered the numerically highest damage with 72.1 ± 2.0 infested shoots per 100 shoots, followed by the medium-shade HNDYZ tea plantation with the 59.5 ± 3.2 infested shoots. Conversely, the numerically lowest damage of *H. theivora* presented in the no-shade HNDYZ tea plantation, which was significantly lower than those in the medium-shade and high-shade HNDYZ tea plantations, respectively (**Figure 9**). The results indicated that shade conditions could influence the feeding damage caused by *H. theivora*, with tea plantations in the higher shade more susceptible to such damage.

## 4 Discussion

The HNDYZ is a typical large-leaf plant and the most common tea cultivar cultivated throughout the tea regions of Hainan, China. Tea mosquito bugs are the most destructive insect pests in HNDYZ tea plantations. Therefore, understanding the feeding activities of *H. theivora* on HNDYZ tea plants can provide valuable evidence for elucidating the ecological adaptability of this pest in the tropical tea regions of South China. In this study, we first described the morphological characteristics of eggs, nymphs and adults of *H. theivora* in Hainan tea region. Consequently, biological characteristics associated with the feeding damage of *H. theivora* on HNDYZ tea shoots were systematically investigated under laboratory and field conditions.

The morphology of *H. theivora* in tea plantations in Hainan is similar to that of identical species described in other tropical tea plantations and various crop habitats, such as cocoa, cashew, and coffee, in India, Bangladesh, Sri Lanka, and Thailand (Roy et al., 2015; Saroj et al., 2016; Sankarganesh et al., 2020; Jakkoksung et al., 2023). The *H. theivora* eggs were mainly deposited within the epidermic tender stem with two unequal respiratory horns exposed. However, the two chorionic processes only exhibited during the egg stage and would leave with the eggshell as the fresh 1st-instar nymph hatched out (Personal observation). The color and body size among the five instar nymphs showed a clear distinction from the young to mature stages. The colors transitioned from nacarat to light green and dark green, while the body sizes gradually increased. This growth corresponded with a larger number of feeding spots and greater acreage. This result is consistent with previous investigations showing the 5th-instar nymph to be the most voracious feeder throughout its life (Bhuyan and Bhattacharyya, 2006). The body size of females *H. theivora* was significantly larger than that of males, and the area of feeding spots produced by females was noticeably larger than those produced by males despite no significant quantitative difference. This suggests that females cause more feeding damage than males on the HNDYZ tea plants. Therefore, more efforts are needed to address the feeding behaviors of *H. theivora*, particularly concerning the 5th-instar nymph and adult female, two crucial stages that require further exploration for integrated pest management strategies in tropical tea plantations.

The suitability of the host HNDYZ tea plant is the primary factor for *H. theivora*’s survival in the Hainan tea region. The tea mosquito bug is a polyphagous pest that attacks multiple cash crops, however, tea is one of the most suitable host plants. Here, nymphs and adults exhibit their preferred feeding and oviposition choices and achieve better survival and reproductive performance (Ahmed and Mamun, 2014; Roy et al., 2015; Vishnupriya et al., 2021). The results of the current study indicated that *H. theiora* preferred to feed on the 2nd and 1st leaves of HNDYZ tea shoot where nymphs and adults could be seen easily and quickly to complete a feeding spot with averagely expending 2 ~ 4 min. Moreover, one 3rd-instar nymph could quantitatively make 69.2 feeding spots per day, while adults produced more feeding spots than nymphs during the feeding peak between 5:00 PM and 1:00 AM. Additionally, the adult females and 5th-instar nymphs could produce average feeding spots on the HNDYZ tea leaves, measuring 5.98 mm² and 5.04 mm², respectively. This suggests that *H. theivora* could effectively settle on the HNDYZ tea cultivar in Hainan, China. It is worth mentioning that the fresh feeding spots on tender tea leaves will shortly turn to dark brown within one day and result in the leaf drying and necrosis in 3 ~ 4 days, which is almost the same as a previous description in the tea growing regions in India (Roy et al., 2015). Considering that the feeding damage caused by tea mosquito bugs is the most destructive to tea plants, further research should be conducted to explore the feeding mechanism of *H. theivora* on HNDYZ tea plants to mitigate this threat.

The adaptability to the tropical climate is another crucial factor for *H. theivora*’s survival in the Hainan tea region. In general, the tea mosquito bug is a typical sucking pest found in tropical environments (Roy et al., 2024). However, in some previous investigations, tea mosquito bugs were identified as a well-adapted species, particularly in the variable climatic conditions of tea plantations (Chakraborty and Chakraborty, 2005; Roy et al., 2009; Pakrashi and Sarma, 2014; Sarma et al., 2014; Wagiman et al., 2021). Hainan is a tropical island located in southern China. Higher temperatures and humidity create ideal environmental conditions for the survival and production of *H. theivora*. Meanwhile, tea mosquito bugs have become the primary pests in most tea plantations in similar tropical climates across India, Sri Lanka, and Bangladesh (Ahmed and Mamun, 2014; Roy et al., 2015; Babu et al., 2023). Although *H. theivora* occurs year-round in the Hainan tea region, the peak infestation in tea plantations occurs from August to November (Meng et al., 2020; Zhou et al., 2023a). Interestingly, this period coincides completely with the monsoon season in Hainan, suggesting that rainfall may be the key factor mediating the occurrence of *H. theivora* in the HNDYZ tea plantations. Additionally, a positive relationship between humidity (i.e., rainfall) and tea mosquito bug infestations has been reported in some tea regions in India (Chakraborty and Chakraborty, 2005; Pakrashi and Sarma, 2014). In this study, we found that the feeding peak of *H. theivora* occurred at night and in the morning when there was little to no sunlight, coinciding with enriched dew on tender tea shoots. Nevertheless, further research is needed to understand how tea mosquito bugs adapt to extreme climates, such as high temperatures, strong winds, and/or rainfall, in the tropical tea plantations of Hainan during the monsoon season.

The feeding preference of tea mosquito bugs on tea plants is influenced by the various tea cultivars, which possess diverse morphological, physiological, and biochemical characteristics in their tender shoots, as well as by the different shade conditions found in tea plantations (Hazarika et al., 2009; Roy et al., 2015). Our field investigations indicated that *H. theivora* preferred feeding on the large-leaf tea cultivar (Hainan Dayezhong tea) rather than the small-leaf tea cultivar (Jinxuan tea). Firstly, the larger leaf size, both in length and width, as well as the thicker tender tissues (mesophyll cells) of HNDYZ tea leaves, supported the nymphs and adults of *H. theivora*, providing them with more opportunities for puncturing and sucking (Chen et al., 2019). Secondly, the varying levels of several distinguished volatile and non-volatile metabolites between large-leaf and small-leaf tea cultivars may constitute crucial components that influence the susceptibility or resistance of these cultivars to insects. These metabolites could affect habitat orientation, as well as the feeding attempts and choices made by *H. theivora* on tea plants (Zhou et al., 2023b; Hu et al., 2024). This strong correlation between the phytochemical profile of tea plants and the infestation of tea mosquito bugs has been reported in several previous studies (Chowdhury et al., 2016; Suganthi et al., 2018; Asmara et al., 2021; Samynathan et al., 2023). Meanwhile, the HNDYZ has been verified as an independently originated tea cultivar, which also contains specialized primary and secondary metabolites (Guo et al., 2024; Gou et al., 2024; Hu et al., 2024). Nevertheless, whether HNDYZ is a susceptible tea cultivar to *H. theivora* and what chemical components might mediate this feeding preference still need more research.

In addition, the results from our field surveys indicated that feeding damage by *H. theivora* was most severe in high-shade tea plantation, followed by medium-shade and no-shade tea plantations. This suggests that shade conditions are a crucial factor influencing the feeding choices of *H. theivora* on HNDYZ tea plants. Undoubtedly, the varying sunlight exposure of tea bushes under different shade conditions alters the morphological, physiological, and biochemical properties of tender shoots during the growth and development of tea plants (Chen et al., 2019). This, in turn, affects the tea mosquito bugs’ habitat and host location before they land on the tea plants, as well as their recognition of feeding sites and feeding decisions after landing and coming into contact with the HNDYZ tender shoots (Knolhoff and Heckel, 2014). HNDYZ is an indigenous tea cultivar planted for hundreds of years in the tropical rainforest districts of Wuzhishan city, Hainan, China (Guo et al., 2024). According to the encouragement tactics of underwood planting for protecting the tropical rainforest in Hainan, different types of plants have been intercropped in the HNDYZ tea plantations. These include the betel palm (*Areca catechu*), the rubber tree (*Hevea brasiliensis*), and several tropical arbor trees (Guo et al., 2006; Peng et al., 2023). Meanwhile, the shade conditions in a tea plantation can be influenced not only by the intercrops and cover crops planted within the plantation but also by the terrain and orientation of the tea plantation (Pokharel et al., 2023; Maleque et al., 2024). However, the effects of different shade conditions on the metabolites of HNDYZ tea shoots have yet to be tested, and further investigations are needed in the future.

Taken together, our current studies describe the morphology of *H. theivora* in HNDYZ tea plantations in Hainan, China. We have investigated and analyzed the feeding biology related to the symptoms observed in feeding plots, including the feeding time and locations, as well as the amounts and areas of feeding on HNDYZ tea shoots under laboratory conditions. Furthermore, we have surveyed and discussed the feeding preferences of *H. theivora* between the large-leaf HNDYZ tea cultivar and the small-leaf Jinxuan tea cultivar, as well as the feeding infestations in three HNDYZ tea fields under different shade conditions. For future research on tea mosquito bugs in the Hainan tea region, further exploration is needed regarding host tea-plant habitat orientation, feeding site search and recognition, feeding attempts and acceptance of tender shoots, and the deeper relationship between tea mosquito bugs and HNDYZ tea plants.

## Data Availability

The raw data supporting the conclusions of this article will be made available by the authors, without undue reservation.

## References

[B1] Ahmed A. Mamun M. S. A. (2014). “Tea mosquito bug, *Helopeltis theivora* Waterhouse (Hemiptera: Miridae): A threat to tea cultivation in Bangladesh,” in Conference: Seminar on Tea Pest and Mosquito Control in Tea Garden Areas of Greater Sylhet Region (Bangladesh Tea Research Institute, Srimangal, Moulvibazar, Bangladesh), 1–11.

[B2] Asmara D. T. Murti R. H. Wijonarko A. Afifah E. N. (2021). Evaluation of resistant tea (*Camellia sinensis L.*) clones against *Helopeltis bradyi* . Agrivita J. Agr. Sci. 43, 518–525. doi: 10.17503/agrivita.v43i3.2557

[B3] Babu A. Roy S. Baruah R. D. Deka B. Ahmed K. Z. Bayen S.. (2023). “Impact of climate change on tea cultivation and adaptation strategies: Special emphasis on tea pests in north east India,” in Climate Change and Agriculture: Perspectives, Sustainability and Resilience. Ed. Benkeblia N. (John Wiley & Sons, New York), 285–310. doi: 10.1002/9781119789789.ch13

[B4] Basnet K. Mukhopadhyay A. (2014). Biocontrol potential of the lynx spider O*xyopes javanus* (Araneae: Oxyopidae) against the tea mosquito bug, *Helopeltis theivora* (Heteroptera: Miridae). Int. J. Trop. Insect Sci. 34, 232–238. doi: 10.1017/S1742758414000538

[B5] Bharathi N. S. Rabeesh T. P. (2023). Seasonal abundance and feeding behaviour of *Oxyopes birmanicu*s Thorell on tea mosquito bug *Helopeltis theivora* Water house. Indian J. Entomol. 86, 451–455. doi: 10.55446/IJE.2023.821

[B6] Bhuyan M. Bhattacharyya P. R. (2006). Feed and oviposition preference of *Helopeltis theivora* (Hemiptera: Miridae) on tea in Northeast India. Insect Sci. 13, 485–488. doi: 10.1111/j.1744-7917.2006.00119.x

[B7] Bordoloi K. S. Baruah P. M. Tanti B. Gill S. S. Agarwala N. (2023). *Helopeltis theivora* responsive transcriptomic reprogramming uncovers long Non-coding RNAs as possible regulators of primary and secondary metabolism in tea plant. J. Plant Growth Regul. 42, 6523–6548. doi: 10.1007/s00344-022-10893-x

[B8] Borthakur S. Bora D. (2023). Identification of chemical cues of *Camellia sinensis* (Ericales: Theaceae) and alternate host plants for preference by tea mosquito bug *Helopeltis theivora* (Hemiptera: Miridae). Int. J. Sci. Res. Arch. 8, 710–719. doi: 10.30574/ijsra.2023.8.1.0123

[B9] Chakraborty U. Chakraborty N. (2005). Impact of environmental factors on infestation of tea leaves by *Helopeltis theivora*, and associated changes in flavonoid flavor components and enzyme activities. Phytoparasitica 33, 88–96. doi: 10.1007/BF02980930

[B10] Chen G. Zhou Y. Zhong S. Wu H. Fu S. Fu R. (2019). Effects of environmental factors on seedling growth of *Camellia sinensis* var. *Assamica* . Horticulture Seed 39, 1–4.

[B11] Chowdhury R. S. Moly I. S. Ahmed M. Mamun M. S. A. Hoque M. M. Miah M. F. (2016). Impact of tea mosquito bug (*Helopeltis theivora*) infestation on the quality of tea (*Camellia sinensis*). Bangladesh J. Zool. 44, 197–207. doi: 10.3329/bjz.v44i2.32759

[B12] Gou Z. Du S. Fu S. Fu M. Fu R. Wu H. (2024). Identification and characteristic analysis of metabolites from the leaves of wild Assam tea (*C. sinensis* var. *assamica*) in Wuzhishan. J. Trop. Biol. 15, 400–406.

[B13] Guo D. Li D. Wang Z. Li D. Zhou Y. Xiang G.. (2024). Genome resequencing reveals an independently originated *Camellia sinensis* variety - Hainan tea. Agrobiodiversity 3, 3–11. doi: 10.48130/abd-0024-0003

[B14] Guo Z. Zhang Y. Deegen P. Uibrig H. (2006). Economic analyses of rubber and tea plantations and rubber-tea intercropping in Hainan, China. Agroforest. Syst. 66, 117–127. doi: 10.1007/s10457-005-4676-2

[B15] Hazarika L. K. Bhuyan M. Hazarika B. N. (2009). Insect pests of tea and their management. Annu. Rev. Entomol. 54, 267–284. doi: 10.1146/annurev.ento.53.103106.093359 19067632

[B16] Hu Y. Wang J. Luo W. Tang J. Tuo Y. Liao N.. (2024). Study on metabolic variation reveals metabolites important for flavor development and antioxidant property of Hainan Dayezhong black tea. Food Res. Int. 196, 115112. doi: 10.1016/j.foodres.2024.115112 39614518

[B17] Jakkoksung A. Attasopa K. Chiu C. I. Chanbang Y. (2023). Life cycle and damage patterns of tea mosquito bug (*Helopeltis theivora* Waterhouse), a newly recorded pest on arabica coffee in northern Thailand. Chiang Mai J. Sci. 50, 1–10. doi: 10.12982/CMJS.2023.047

[B18] Knolhoff L. M. Heckel D. G. (2014). Behavioral assays for studies of host plant choice and adaptation in herbivorous insects. Annu. Rev. Entomol. 59, 263–278. doi: 10.1146/annurev-ento-011613-161945 24160429

[B19] Lu X. Zhang Y. (2023). “Agriculture,” in Hainan Statistical Yearbook 2023. Eds. Yang L. Liu F. (China Statistics Press, Beijing), 207–244.

[B20] Maleque M. A. Ferdous J. Shitel A. A. Ahmed J. Islam A. F. M. S. Mondal M. F.. (2024). Role of shade trees in conserving beneficial arthropods of biocontrol importance in tea ecosystem. Agroforest Syst. 98, 21–36. doi: 10.1007/s10457-023-00886-4

[B21] Meng Z. Li S. Yang W. Zhou Y. (2020). Tea mosquito bug *Helopeltis theivora* . China Tea 42, 17–20.

[B22] Muraleedharan M. (1992). “Pest control in Asia,” in Chapter 12 in Tea: Cultivation to Consumption. Eds. Willson K. C. Clifford M. N. (Springer, Dordrecht), 375–412. doi: 10.1007/978-94-011-2326-6_12

[B23] Pakrashi D. Sarma K. K. (2014). Impact of rainfall intensity on infestation level of tea mosquito bug in tea gardens of north east India – A preliminary study. J. Environ. Manage. 1, 42–48.

[B24] Peng W. Wang Y. Zhou Y. (2023). Research on the development of the *Camellia assamica* industry based on regional characteristics - Taking Wuzhi mountain in Hainan as an example. Chin. J. Trop. Agr. 43, 109–116.

[B25] Pokharel S. S. Yu H. Fang W. Parajulee M. N. Chen H. (2023). Intercropping cover crops for a vital ecosystem service: A review of the biocontrol of insect pests in tea agroecosystems. Plants 12, 2361. doi: 10.3390/plants12122361 37375986 PMC10304037

[B26] Radhakrishnan B. (2022). “Pests and their management in tea,” in Chapter 64 in Trends in Horticultural Entomology. Ed. Mani M. (Springer Nature, Singapore), 1489–1511. doi: 10.1007/978-981-19-0343-4_64

[B27] Roy S. Mukhapadhyay A. Gurusubramanian G. (2009). Varietal preference and feeding behaviour of tea mosquito bug (*Helopeltis theivora* Waterhouse) on tea plants (*Camellia sinensis*). Acad. J. Entomol. 2, 1–9.

[B28] Roy S. Muraleedharan N. Mukhapadhyay A. Handique G. (2015). The tea mosquito bug, *Helopeltis theivora* Waterhouse (Heteroptera: Miridae): its status, biology, ecology and management in tea plantations. Int. J. Pest Manage. 61, 179–197. doi: 10.1080/09670874.2015.1030002

[B29] Roy C. Naskar S. Ghosh S. Rahaman P. Mahanta S. Sarkar N.. (2024). Sucking pest management in tea (*Camellia sinensis* (L.) Kuntze) cultivation: Integrating conventional methods with bio-control strategies. Crop Prot. 183, 106759. doi: 10.1016/j.cropro.2024.106759

[B30] Samynathan R. Venkidasamy B. Shanmugam A. Khaled J. M. Chung I. M. Thiruvengadam M. (2023). Investigating the impact of tea mosquito bug on the phytochemical profile and quality of Indian tea cultivars using HPLC and LC-MS-based metabolic profiling. Ind. Crop Prod. 204, 117278. doi: 10.1016/j.indcrop.2023.117278

[B31] Sankarganesh E. Lavanya S. B. Rajeshwaran B. Mounika M. N. (2020). Tea mosquito bug (*Helopeltis* spp.) - A pest of economically important fruit and plantation crops: Its status and management prospects. J. Plant Health Iss. 1, 014–024.

[B32] Sarma K. K. Pakrasi D. Sudhakar S. (2014). Impact of land use and land cover on infestation level of tea mosquito bug (*Helopeltis theivor*a Waterhouse) in tea garden - A RS&GIS approach. Int. J. Adv. Earth Environ. Sci. 2, 1–11.

[B33] Saroj P. L. Bhat P. S. Srikumar K. K. (2016). Tea mosquito bug (*Helopeltis* spp.) - A devastating pest of cashew plantations in India: A review. Indian J. Agr. Sci. 86, 151–162. doi: 10.56093/ijas.v86i2.55868

[B34] Srikumar K. K. Sukumaran S. Kumar B. S. Radhakrishnan B. (2017). Life history and functional response of two species of Reduviids (Hemiptera: Reduviidae: Harpactorinae) in tea. J. Agr. Urban Entomol. 33, 44–56. doi: 10.3954/JAUE16-17.1

[B35] Suganthi M. Arvinth S. Senthilkumar P. (2018). Defensive responses in tea (*Camellia sinensis*) against tea mosquito bug infestation. Res. J. Biotech. 13, 7–11.

[B36] Vishnupriya R. Yamuna G. Yamuna R. (2021). *Helopeltis theivora* (Tea mosquito Bug) a major pest in tea crop - A review. Gradiva Rev. J. 7, 161–169.

[B37] Wagiman F. X. Sari N. M. Wijonarko A. (2021). The population structure and presence of *Helopeltis bradyi* on the tea plant parts at various times during the day. IOP Conf. Series: Earth Environ. Sci. 686, 12062. doi: 10.1088/1755-1315/686/1/012062

[B38] Wang J. (1985). A study of the biology character and prevent and elimination of the H*elopeltis fasctaticollis* Poppius in the tea trees. Nat. Sci. J. Hainan Univ. 3, 23–31.

[B39] Zhou Y. He W. He Y. Chen Q. Gao Y. Geng J.. (2023b). Formation of 8-hydroxylinalool in tea plant *Camellia sinensis* var. *Assamica* ‘Hainan dayezhong’. Food Chem.: Mol. Sci. 6, 100173. doi: 10.1016/j.fochms.2023.100173 PMC10240414 37284067

[B40] Zhou Y. Zhao D. Ning G. Cui J. Yang L. (2023a). Investigation of diseases and pests affecting Hainan large-leaf tea. China Plant Prot. 43, 30–35.

[B41] Zhu X. (2021). Biological characteristics and toxicity determination of insecticide from *Helopeltis theivora* Waterhouse (Haikou, China: Hainan University).

